# A novel non-sequencing approach for rapid authentication of Testudinis Carapax et Plastrum and Trionycis Carapax by species-specific primers

**DOI:** 10.1098/rsos.172140

**Published:** 2018-04-18

**Authors:** Huan Yang, Pingtian Yu, Yi Lu, Zhaoqun Jiao, Liqun Chen, Ying Zhou, Yuping Shen, Xiaobin Jia

**Affiliations:** 1 School of Pharmacy, Jiangsu University, Zhenjiang 212013, People's Republic of China; 2 School of Biological Sciences, Nanyang Technological University, 637551 Singapore; 3 School of Traditional Chinese Medicine, China Pharmaceutical University, Nanjing 211198, People's Republic of China

**Keywords:** species-specific primer, authentication, animal-derived Chinese medicine, Testudinis Carapax et Plastrum, Trionycis Carapax, polymerase chain reaction

## Abstract

A novel non-sequencing approach was developed to detect short DNA fragments (*ca* 100 bp) for rapid authentication of two natural products, namely Testudinis Carapax et Plastrum and Trionycis Carapax, based on the difference in mitochondrial genome. Five specifically designed primer reactions were established to target species for reliable identification of their commercial products. They were confirmed to have a high level of inter-species-specificity and good intra-species stability. The limit of detection was estimated to be 1 ng of genomes for all of five assays. Also, the validation results demonstrated that the raw materials and processed products in addition to some of the highly processed products can be conveniently authenticated with good sensitivity and precision by this newly proposed approach. Especially, when reference sample mixtures were assayed, these primer sets have still performed well but not the prevailing COI barcoding technology. These could assist in the discrimination and identification of other animal-derived medicines for their form of raw material, the pulverized and the complex.

## 1. Introduction

Testudinis Carapax et Plastrum (TCP) and Trionycis Carapax (TC) are derived from the shell of *Chinemys reevesii* and *Pelodiscus sinensis*. They are not only important components in Chinese patent medicines (CPMs), but also are used for health supplements or functional foods. CPMs composed of TCP have a well-recognized curative effect in the treatment of osteoporosis, diabetic nephropathy, hypo-immunity, ageing, insomnia with sweating at night, etc. [1–3]. In addition, TC is an integral part of CPMs that are beneficial to cure cirrhosis, hepatitis, tuberculosis, Yin deficiency, as well as to inhibit tumour growth [4–6].

However, there are also numerous reports of fake or adulterant Chinese medicine, which has exposed consumers to a major public health risk and caused a non-negligible drug market disorder. Adulteration through the addition or substitution of similar substances in traditional Chinese medicine (TCM) is a type of medicine fraud defined as the intentional fraudulent modification of medicines to obtain a financial advantage. The fact that these two reptiles can be used for medicinal purpose only after their ages are above 3 years has caused an increasing scarcity of resources and economically motivated adulteration. On the other hand, similar morphological characteristics among species of close phylogenetic relationship and lack of professional experience can frequently lead to unintentional use of fake products. Moreover, both TCP and TC are processed in a manner where cutting, heating and sometimes even addition of vinegar are involved.

Accurate authentication of Chinese medicines is a strict legal requirement in many countries throughout America, Europe and Asia, and is a prerequisite for delivering a quality product that meets consumer expectations. Many efforts have been made to identify the origin of animal species in medicines and foods. Fourier transform infrared (FTIR), high-performance liquid chromatography (HPLC) and mass spectrometry (MS) have been employed to differentiate among species by spectral intensity and chromatographic behaviour, but similar chemical properties always make accurate identification of a mixture difficult [7–11]. Enzyme-linked immunosorbent assay (ELISA) is well recognized as a sensitive and robust technique for detecting low levels of original material from a species based on antibody and antigen reactions; however, species-specificity can be significantly compromised by high homology of protein sequences among animals and a high concentration of salt [12,13]. In recent decades, polymerase chain reaction (PCR)-based methods to verify the origin of a species have been considered the most preferred technology owing to the favourable specificity and stability of DNA fragments. For instance, the genome of ancient Egyptian mummies was used to research ancient human history and offered the perspective of deciphering Egypt's past [14]. Furthermore, DNA is present in most biological tissues and can be readily extracted from even a very small amount of test sample. Therefore, PCR-based methods are an ideal and powerful tool for identification of original material existing in final products [15,16].

DNA barcoding, particularly cytochrome *c* oxidase subunit 1 gene (COI) barcoding, has for a long time been most often used as an important means to identify ingredients and to detect spurious species, such as Cervi Cornu Pantotrichum (deer horn) and Serpentis Periostracum (snake slough) [17–19]. However, this method requires high purity of a DNA sample and cannot be applied to a mixture, such as adulterated products, or even medicinal materials slightly contaminated by other species. Moreover, COI universal DNA primers amplify a 710-bp fragment of the mitochondria, but the DNA from processed products is often severely degraded into very short fragments. So, for the analysis of these samples, the latter is a much more preferred target than the former. Hellebrand *et al.* [20] studied the influence of amplification length on test results, and it was found that the amplification of short fragments is more successful than that of longer fragments. On the other hand, intraspecific variation threshold is ambiguous owing to many factors including the interference of fluorescent dyes, base mismatching, evolutionary rate and so on. The divergence in a few cnidarians was far less than that typical for other animal phyla [21]. However, new primers designed to bind to highly conserved gene regions upstream of COI will aid the amplification of this gene region in species where standard primers fail, and will provide valuable information [13]. It is suggested that the threshold for intraspecific variation determined may be anything but convincing owing to various evolutionary rates and loci. Moreover, COI barcoding technology is of low capability in disturbance rejection, and it always undergoes a sequencing procedure of amplicon after PCR amplification. Accordingly, it was not commonly recommended that universal primers are used in DNA barcoding of processed material of Chinese medicine for species identification.

**Figure 1.**
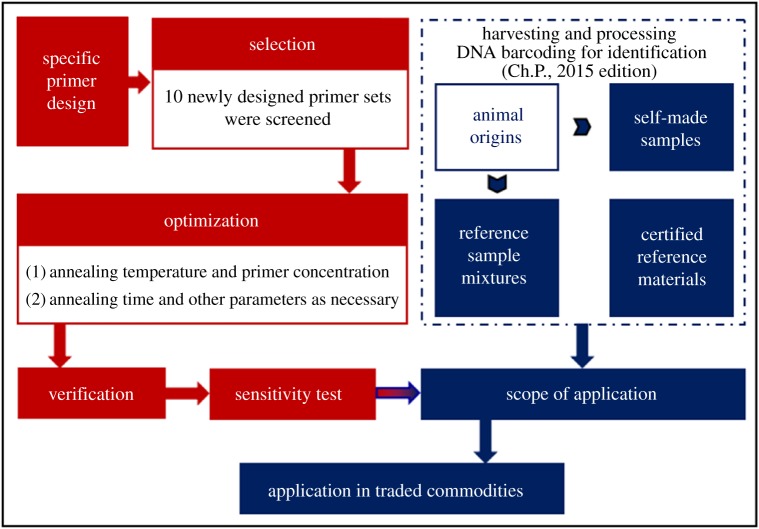
Flow chart for the establishment of the novel strategy.

Mitochondrial DNA is applied for species identification because there are multiple copies per cell. The mitochondrial genome (mitogenome) in vertebrates consists of a circular DNA sequence of approximately 16–18 kb containing one control region, 22 tRNA sequences, two rRNA sequences and 13 peptide coding genes [22]. But the conservative areas of the species sometimes are not necessarily conserved in different individuals of the same species, and this has been leading to the failure of PCR amplification in some applications. Therefore, in this study, species-specific primers are particularly designed according to both intraspecific homology and interspecific variation in the mitochondrial complete genome of TCP, TC and their similar species. The aim was to establish a novel non-sequencing and reliable PCR-based approach that can be used for specific and rapid authentication of TCP and TC (figure 1). In addition, these could also assist in the discrimination and identification of adulterants of other animal-derived Chinese medicines for their form of raw medicinal material, the pulverized and even the complex.

## 2. Experimental set-up

### 2.1. Materials

**Figure 2.**
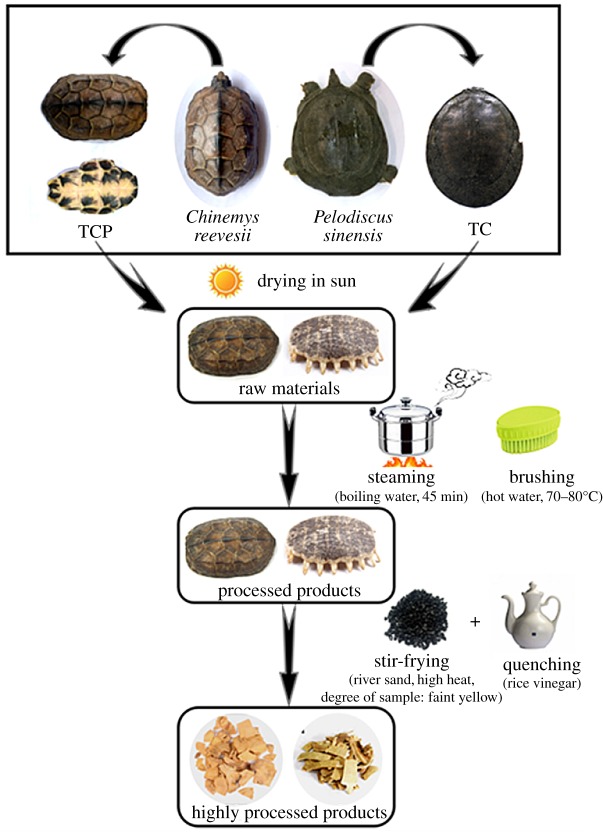
Procedures for self-made raw materials and processed products.

Three Testudinidae species including *Chinemys reevesii*, *Trachemys scripta* and *Mauremys sinensis*, and two Trionychidae species including *Pelodiscus sinensis* and *Apalone ferox*, were used in this study. Collection locations of these animals are listed in table 1, and all of the original samples were verified by COI barcoding. The tortoises and turtles were then handled to prepare the raw materials and processed products of their carapaces according to the relevant protocols recorded in the prevailing China Pharmacopoeia (Ch.P., 2015 edition) [23], as illustrated in figure 2.

**Table 1.** Testudinidae and Trionychidae species used in the study.

code	species	collection site	collection date	code	species	collection site	collection date
CR1	*Chinemys reevesii*	Huzhou, Zhejiang, PRC	21 July 2016	PS1	*Pelodiscus sinensis*	Wuxi, Jiangsu, PRC	27 Aug 2016
CR2	*Chinemys reevesii*	Huzhou, Zhejiang, PRC	21 July 2016	PS2	*Pelodiscus sinensis*	Yancheng, Jiangsu, PRC	26 Aug 2016
CR3	*Chinemys reevesii*	Shanghai, PRC	21 July 2016	PS3	*Pelodiscus sinensis*	Yancheng, Jiangsu, PRC	26 Aug 2016
CR4	*Chinemys reevesii*	Wuxi, Jiangsu, PRC	27 Aug 2016	PS4	*Pelodiscus sinensis*	Taizhou, Jiangsu, PRC	28 Aug 2016
CR5	*Chinemys reevesii*	Wuxi, Jiangsu, PRC	27 Aug 2016	PS5	*Pelodiscus sinensis*	Taizhou, Jiangsu, PRC	28 Aug 2016
CR6	*Chinemys reevesii*	Yancheng, Jiangsu, PRC	25 Aug 2016	PS6	*Pelodiscus sinensis*	Nanyang, Henan, PRC	26 Aug 2016
CR7	*Chinemys reevesii*	Zhuji, Zhejiang, PRC	4 Sep 2016	PS7	*Pelodiscus sinensis*	Nanyang, Henan, PRC	30 Aug 2016
CR8	*Chinemys reevesii*	Zhenjiang, Jiangsu, PRC	28 Aug 2016	PS8	*Pelodiscus sinensis*	Jianyang, Sichuan, PRC	29 Aug 2016
CR9	*Chinemys reevesii*	Hangzhou, Zhejiang, PRC	2 Sep 2016	PS9	*Pelodiscus sinensis*	Zhenjiang, Jiangsu, PRC	2 Sep 2016
CR10	*Chinemys reevesii*	Nanyang, Henan, PRC	26 Aug 2016	PS10	*Pelodiscus sinensis*	Zhenjiang, Jiangsu, PRC	12 Nov 2016
TS1	*Trachemys scripta*	Zhenjiang, Jiangsu, PRC	14 May 2016	AF1	*Apalone ferox*	Suzhou, Jiangsu, PRC	7 Sep 2016
TS2	*Trachemys scripta*	Wuxi, Jiangsu, PRC	27 Aug 2016	AF2	*Apalone ferox*	Suzhou, Jiangsu, PRC	7 Sep 2016
MS1	*Mauremys sinensis*	Hangzhou, Zhejiang, PRC	16 May 2016				
MS2	*Mauremys sinensis*	Wuxi, Jiangsu, PRC	27 Aug 2016				

A total number of 64 commercial samples including raw materials, processed products and highly processed products were collected for species identification using the newly proposed approach. Three forms of traded commodities were collected from the TCM wholesale market, manufacturers and TCM hospitals as detailed in table 2.

**Table 2.** Traded commodities tested in the study.

code	product	batch no.	source	code	product	batch no.	source
MG1	raw TCP	JM17050401	Jiu Ming Tang, Bozhou, Anhui, PRC	MB1	raw TC	JM17050401	Jiu Ming Tang, Bozhou, Anhui, PRC
MG2	raw TCP	JM17050402	Jiu Ming Tang, Bozhou, Anhui, PRC	MB2	raw TC	JM17050402	Jiu Ming Tang, Bozhou, Anhui, PRC
MG3	raw TCP	JM17050403	Jiu Ming Tang, Bozhou, Anhui, PRC	MB3	raw TC	JM17050403	Jiu Ming Tang, Bozhou, Anhui, PRC
MG4	raw TCP	JM17050904	Jiu Ming Tang, Bozhou, Anhui, PRC	MB4	raw TC	NY17050501	Ning Yun Tang, Bozhou, Anhui, PRC
MG5	raw TCP	JM17050905	Jiu Ming Tang, Bozhou, Anhui, PRC	MB5	raw TC	NY17050502	Ning Yun Tang, Bozhou, Anhui, PRC
MG6	raw TCP	JM17050906	Jiu Ming Tang, Bozhou, Anhui, PRC	MB6	raw TC	QZ17050401	Qi Zhou Tang, Baoding, Hebei, PRC
MG7	raw TCP	NY17050501	Ning Yun Tang, Bozhou, Anhui, PRC	MB7	raw TC	QZ17050402	Qi Zhou Tang, Baoding, Hebei, PRC
MG8	raw TCP	NY17050502	Ning Yun Tang, Bozhou, Anhui, PRC	MB8	raw TC	QZ17050403	Qi Zhou Tang, Baoding, Hebei, PRC
MG9	raw TCP	QZ17050401	Qi Zhou Tang, Baoding, Hebei, PRC	MB9	raw TC	QZ17050404	Qi Zhou Tang, Baoding, Hebei, PRC
MG10	raw TCP	QZ17050402	Qi Zhou Tang, Baoding, Hebei, PRC	MB10	raw TC	QZ17050405	Qi Zhou Tang, Baoding, Hebei, PRC
RG1	processed TCP	160101	Bai Shi Xing, Bozhou, Anhui, PRC	RB1	processed TC	160105	Bai Shi Xing, Bozhou, Anhui, PRC
RG2	processed TCP	160201	Bai Shi Xing, Bozhou, Anhui, PRC	RB2	processed TC	160201	Bai Shi Xing, Bozhou, Anhui, PRC
RG3	processed TCP	160301	Bai Shi Xing, Bozhou, Anhui, PRC	RB3	processed TC	160301	Bai Shi Xing, Bozhou, Anhui, PRC
RG4	processed TCP	160401	Bai Shi Xing, Bozhou, Anhui, PRC	RB4	processed TC	160401	Bai Shi Xing, Bozhou, Anhui, PRC
RG5	processed TCP	160501	Bai Shi Xing, Bozhou, Anhui, PRC	RB5	processed TC	160501	Bai Shi Xing, Bozhou, Anhui, PRC
RG6	processed TCP	160601	Bai Shi Xing, Bozhou, Anhui, PRC	RB6	processed TC	160701	Bai Shi Xing, Bozhou, Anhui, PRC
RG7	processed TCP	160801	Bai Shi Xing, Bozhou, Anhui, PRC	RB7	processed TC	160801	Bai Shi Xing, Bozhou, Anhui, PRC
RG8	processed TCP	161001	Bai Shi Xing, Bozhou, Anhui, PRC	RB8	processed TC	160901	Bai Shi Xing, Bozhou, Anhui, PRC
RG9	processed TCP	161101	Bai Shi Xing, Bozhou, Anhui, PRC	RB9	processed TC	161001	Bai Shi Xing, Bozhou, Anhui, PRC
RG10	processed TCP	170101	Bai Shi Xing, Bozhou, Anhui, PRC	RB10	processed TC	161101	Bai Shi Xing, Bozhou, Anhui, PRC
PG1	highly processed TCP	160105	Bai Shi Xing, Bozhou, Anhui, PRC	PB1	highly processed TC	160101	Bai Shi Xing, Bozhou, Anhui, PRC
PG2	highly processed TCP	160201	Bai Shi Xing, Bozhou, Anhui, PRC	PB2	highly processed TC	160201	Bai Shi Xing, Bozhou, Anhui, PRC
PG3	highly processed TCP	160301	Bai Shi Xing, Bozhou, Anhui, PRC	PB3	highly processed TC	160301	Bai Shi Xing, Bozhou, Anhui, PRC
PG4	highly processed TCP	160401	Bai Shi Xing, Bozhou, Anhui, PRC	PB4	highly processed TC	160401	Bai Shi Xing, Bozhou, Anhui, PRC
PG5	highly processed TCP	160501	Bai Shi Xing, Bozhou, Anhui, PRC	PB5	highly processed TC	160501	Bai Shi Xing, Bozhou, Anhui, PRC
PG6	highly processed TCP	160601	Bai Shi Xing, Bozhou, Anhui, PRC	PB6	highly processed TC	160601	Bai Shi Xing, Bozhou, Anhui, PRC
PG7	highly processed TCP	160701	Bai Shi Xing, Bozhou, Anhui, PRC	PB7	highly processed TC	160801	Bai Shi Xing, Bozhou, Anhui, PRC
PG8	highly processed TCP	160801	Bai Shi Xing, Bozhou, Anhui, PRC	PB8	highly processed TC	160901	Bai Shi Xing, Bozhou, Anhui, PRC
PG9	highly processed TCP	161001	Bai Shi Xing, Bozhou, Anhui, PRC	PB9	highly processed TC	161001	Bai Shi Xing, Bozhou, Anhui, PRC
PG10	highly processed TCP	161101	Bai Shi Xing, Bozhou, Anhui, PRC	PB10	highly processed TC	170101	Bai Shi Xing, Bozhou, Anhui, PRC
PG11	highly processed TCP	151208	Shang yao, Yixing, Jiangsu, PRC	PB11	highly processed TC	161011	Weibo, Bozhou, Anhui, PRC
PG12	highly processed TCP	141101	Ruicao, Bozhou, Anhui, PRC	PB12	highly processed TC	161026	Huahong, Danyang, Jiangsu, PRC

### 2.2. DNA extraction

All solid samples were ground into their fine powder, and then subject to genomic DNA extraction by SDS-based protocols. In detail, 50 mg of the homogenized sample was mixed with 995 µl of extraction buffer (100 mM NaCl, 10 mM Tris–HCl (pH 8.0), 25 mM EDTA, 0.5% (w/v) SDS) and 5 µl proteinase K (20 mg ml^−1^), and the mixture was incubated at 56°C for 6 h. After centrifugation at 12 000 r.p.m. for 15 min, 800 µl of the supernatant was transferred to a new clear tube. An equal volume of Tris–phenol solution, PCI solution and CI solution were sequentially mixed with the supernatant for further purification. Then, 450 µl of the supernatant was precipitated by 900 µl of 96% ethanol and 45 μl 5.0 M KAc. The supernatant was removed after centrifugation at 12 000 r.p.m. for 15 min, and the resulting DNA pellet was washed with 70% ethanol and finally reconstituted in 25 µl of TE buffer (pH 8.0) for subsequent experiments.

Nucleic acid and protein spectrophotometry (BioSpec-mini, Shimadzu) were used to quantify the purity and concentration of the extracted DNA. These DNA samples extracted from raw materials or processed products were diluted to 50 ng µl^−1^, and those from highly processed products were used directly as template in further PCR assays.

### 2.3. Target gene selection and primers design

**Figure 3.**
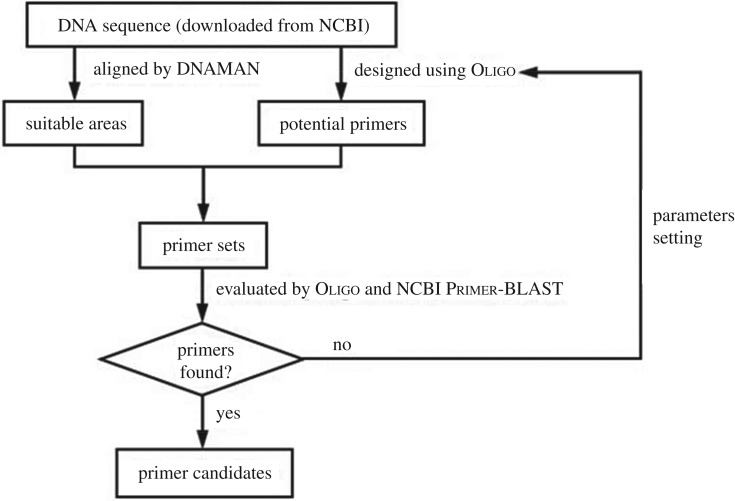
Flowchart for the design of species-specific primer sets.

Fifteen mitochondrial genome sequences of five species, including *Chinemys reevesii* (Accession No.: NC_006082.1, AY676201.1, FJ469674.1, KJ700438.1), *Trachemys scripta* (Accession No.: NC_011573.1, FJ392294.1, KM216749.1), *Mauremys sinensis* (Accession No.: NC_016685.1, FJ871126.1, KC333650.1), *Pelodiscus sinensis* (Accession No.: NC_006132.1, AY687385.1, AY962573.1) and *Apalone ferox* (Accession No.: NC_014054.1, FJ890514.1), were used as targets. SuiTab. areas for designing species-specific primers were identified by DNAMAN software (v. 8.0.8.789), and species-specific primers for *Chelonia* species identification were then designed using Oligo software (v. 7.60). Primer sets were evaluated by Oligo and online NCBI Primer-BLAST (figure 3). All the primers were then synthesized by Sangon Biotech (Shanghai) Co., Ltd. Species-specific primer sets and their characteristics are summarized in table 3. Specificity testing with each primer set in the PCR assays was performed against five selected samples (table 1).

**Table 3.** Primer sets used for PCR assay in this study.

species	code		sequence (5′–3′)	target gene	amplicon size (bp)
*Chinemys reevesii*	PCR-1	F	TATCGTTACAGCCCATGCCT	COX1	101
		R	GCGCTCCGATCATTAAAGGT		
	PCR-2	F	AACCTGGCATATTATGGTCT	D-LOOP	120
		R	CAATCAACTTGAACGAGGGT		
*Trachemys scripta*	PTS-1	F	AGAGAAGGACTTTAACCCTCG	tRNAPro	87
		R	GTTTATGCCCGATAGACCTCA		
	PTS-2	F	GCCCAAACTAACAGACAACCG	12S rRNA	81
		R	CAGCGAAGTAAGTAGTTCACC		
*Mauremys sinensis*	PMS-1	F	TCCTCGGGATAATCCACGAAC	ND6	105
		R	CCATGGCTTTATCGTCTTGGT		
	PMS-2	F	TGTCACCTATTACGCTGGCAA	COX1	90
		R	ACAATAAAGCCCAGGAAACCG		
*Pelodiscus sinensis*	PPS-1	F	AGCCCTATCAGTTTGAATACCAC	ND1	120
		R	CAACCGGACCATATAATTGAGT		
	PPS-2	F	ATATGACTACTAGCCGCACT	ND4	120
		R	GGCAGCTAAGATTATTGACCC		
*Apalone ferox*	PAF-1	F	ATTAGCCACACTACACGGAGGA	COX1	89
		R	TTAAGCCTCCAATGGTTCCGAA		
	PAF-2	F	CCTATCACTACACCCCATACAAC	ND4L	114
		R	ATGCTACTAATAATGACACCCC		

### 2.4. PCR amplification and DNA sequencing

PCR amplification was carried out in a final reaction volume of 25 µl composed of 2.5 µl 10 × PCR buffer, 2.0 mM MgCl_2_, 0.2 mM of each dNTP, approximately 0.2--0.4 µM of each primer, approximately 0.625--1 unit Taq polymerase, 19.875 µl ultrapure water and 1 µl DNA template. The PCR cycler conditions used were an initial denaturation at 95°C for 3 min, followed by approximately 30--35 cycles of 95°C for 30 s, approximately 60--68°C for 30 s and 72°C for 1 min with a final extension at 72°C for 7 min. After resolution by 3% agarose gel electrophoresis and staining in ethidium bromide, the resulting amplicons were visualized under UV light. In order to verify the sequences of short-length fragments produced by the species-specific primers, PCR products were subjected to sequencing in both directions by Sangon Biotech (Shanghai) Co., Ltd.

### 2.5. Specificity and sensitivity

The verification of specificity was carried out under the optimum conditions for different batches of five *Chelonia* species. Sensitivity of the selected species-specific primers was determined in a concurrent PCR run with DNA template of a series of concentrations (10, 1, 0.1 and 0.01 ng µl^−1^) while the primer remained unchanged.

### 2.6. Analysis of reference sample mixtures

Prior to mixing to create reference carapace mixtures, samples of different species were collected to undergo DNA extraction and serve as a positive control. Seven reference carapace mixtures (CR : PS, CR : TS, CR : MS, CR : AF, PS : AF, PS : TS and PS : MS) were prepared at five levels (7 : 1, 3 : 1, 1 : 1, 1 : 3, 1 : 7) of one species mixed with the second species, with a total weight of 50 mg per sample. Individual samples were homogenized with 1 ml of SDS extraction buffer for DNA extraction by SDS-based protocols as aforementioned. Then, the selected primers and the optimized PCR conditions were applied to these reference samples.

### 2.7. Analysis of self-made samples and certified reference material

To investigate the scope of application of the newly proposed approach, 26 raw materials, 26 processed products, 26 highly processed products and a certified reference material of TCP (code: P; B/N: 121494-201604, National Institutes for Food and Drug Control, PRC) were analysed. The PCR assay was performed under optimized conditions using the species-specific primer sets.

### 2.8. Application of PCR assay to commercial products

As a next step, the developed method was used to assess the authenticity of 64 commercially available products, including both TCP and TC varieties, for the identification of animal origins and the verification of labelling compliance. Fifty milligrams of these samples were individually subjected to DNA extraction, and the optimized PCR conditions for each species were then applied to the sample extracts.

## 3. Results and discussion

### 3.1. Screening of primer sets

**Figure 4.**
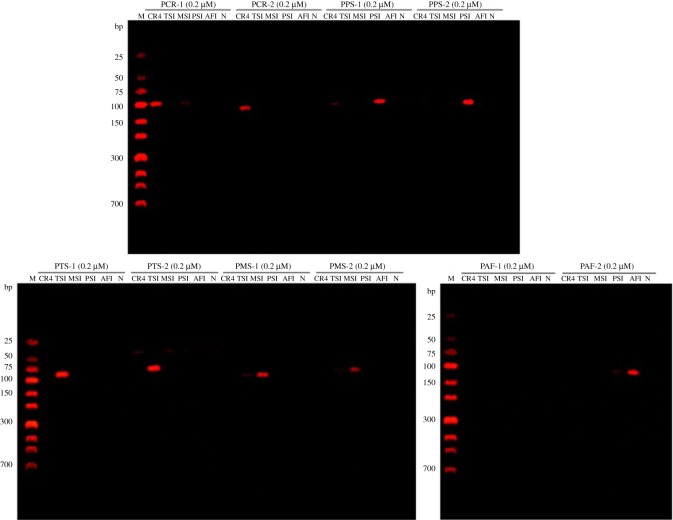
Screening of designed primer sets for species-specificity (M: Low ladder, SN127).

Mitochondrial complete gene sequences from five species of *Chelonia* were incorporated to develop an accurate and rapid method for their identification. The specificity tests of the designed primers were predicted by the Primer-BLAST tool (table 4) and performed by uniplex PCR assay (figure 4). When the primers were used for PCR amplification of genomic DNA extracted from five *Chelonia* species, PAF-1 does not amplify the target gene, and the designed primer sets except for PTS-1 and PTS-2 showed faint false-positive amplification at the corresponding location for the individual species. This false-positive amplification might be caused by improper PCR conditions. Consequently, PCR-2, PTS-2, PMS-1, PPS-2 and PAF-2, which resulted in stronger intensity bands without visible false-positive amplification, were selected for subsequent optimization of PCR conditions.

**Table 4.** Primers used for PCR predicted by Primer-BLAST tool. N, no target templates were found in selected database: Nucleotide collection (nt). (Organism limited to designated species).

	species				
code	*Chinemys reevesii*	*Trachemys scripta*	*Mauremys sinensis*	*Pelodiscus sinensis*	*Apalone ferox*
PCR-1	0	N	N	N	N
PCR-2	0	4	N	N	N
PTS-1	N	0	N	N	N
PTS-2	N	0	N	N	N
PMS-1	7	N	0	N	N
PMS-2	2	N	0	N	N
PPS-1	N	N	N	0	N
PPS-2	N	N	N	0	N
PAF-1	N	N	N	N	0
PAF-2	N	N	N	N	0

### 3.2. Optimized PCR conditions

**Figure 5.**
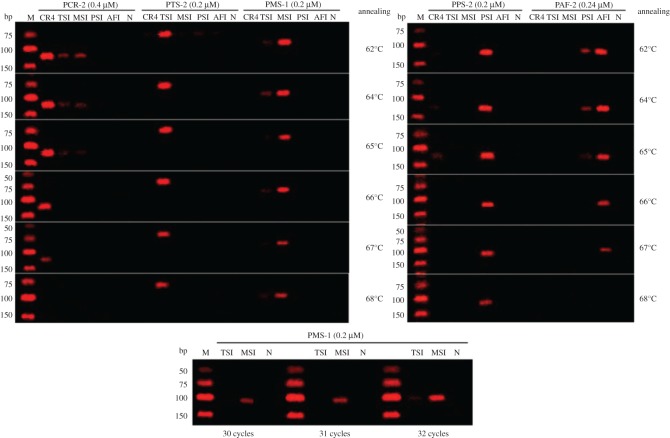
Optimized PCR conditions for five species.

Five primer sets were selected for the reliable identification of five *Chelonia* species and are listed in table 4. The effects of reaction conditions were studied, including the concentration of template and primer, the type and amount of polymerase, annealing temperature and time, cycle times, temperature control method (two or three step) and the performance of equipment (data not shown). PCR conditions were optimized for the five species analysed (figure 5). The PCR cycler conditions used are summarized in table 5.

**Table 5.** Optimized PCR conditions for the five species analysed in this study.

	primers				
programme step	PCR-2	PTS-2	PMS-1	PPS-2	PAF-2
amplification					
initial denaturation	95°C (3 min)^a^				
denaturation	95°C (30 s)^a^				
annealing	66°C (30 s)^a^				
extension	72°C (1 min)^a^				
cycle number	35	35	31	35	35
final extension	72°C (7 min)^a^				

^a^These conditions were the same for all primers.

### 3.3. Specificity and sensitivity

**Figure 6.**
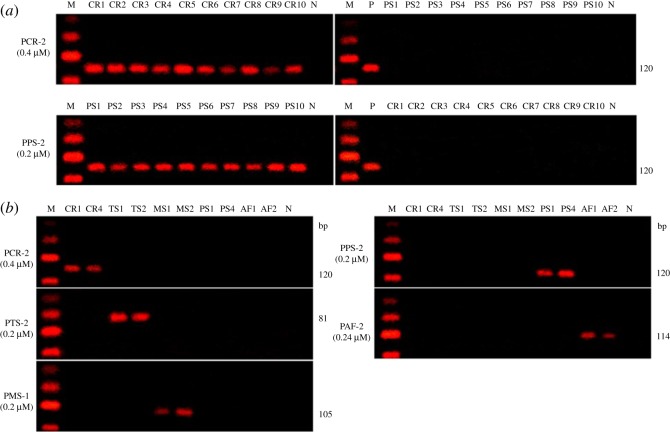
Gels from PCR reactions validating the specificity of five selected primer pairs. (*a*) Specific primers for medicinal ingredients; (*b*) specific primers for non-medicinal ingredients.

**Figure 7.**
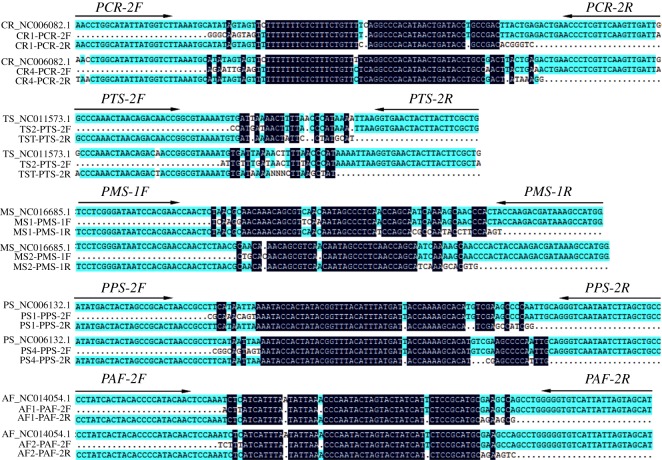
Amplicons of selected primer sets sequenced and aligned.

**Figure 8.**
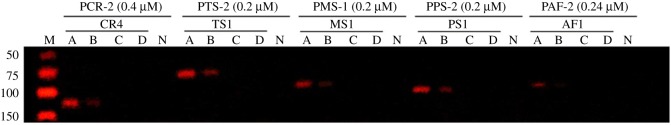
Sensitivity test for five specific primer sets. The concentration of template DNA from lane A to lane D was 10, 1, 0.1 and 0.01 ng µl^−1^.

The verification results of different batches of these five *Chelonia* species clearly demonstrated that each primer set produced a species-specific band without any visible non-specific bands (figure 6). The amplicons were sequenced and edited to verify by BLASTn searches against the GenBank database (figure 7). These primer sets were designed to identify different *Chelonia* species regardless of life stage. Sensitivity of a selected specific primer set for each of the five species was determined using one sample from each species. In all of the species, DNA concentrations of 10 and 1 ng µl^−1^ resulted in strong intensity bands (figure 8).

### 3.4. Analysis of reference sample mixtures

**Figure 9.**
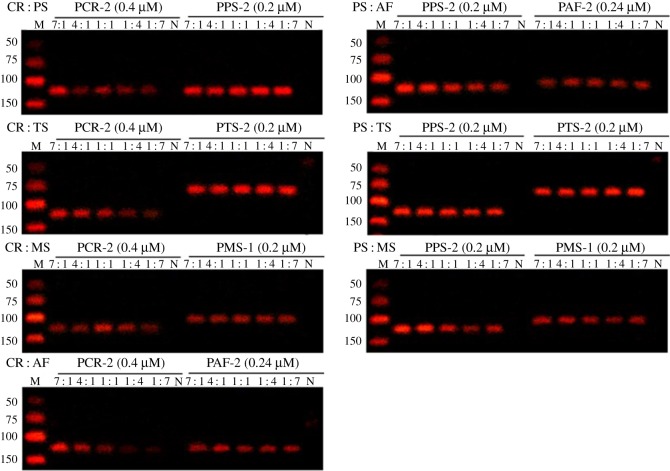
Analysis of reference sample mixtures by PCR.

To investigate whether the selected primers were applicable for adulterated products, 35 reference samples with known ingredient compositions were tested. As shown in figure 9, when these samples were analysed via PCR using selected specific primers, the corresponding species were successfully detected.

### 3.5. Analysis of self-made samples and certified reference material

**Figure 10.**
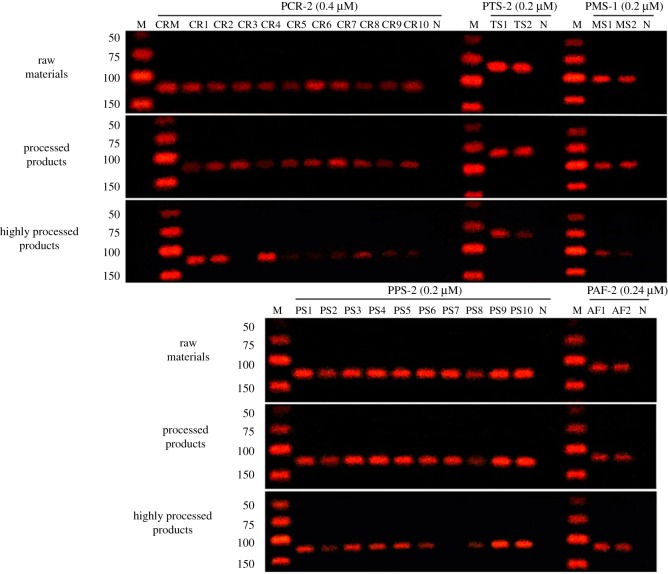
Analysis of self-made samples.

The selected primers were applied to determine their scope in self-made samples and a certified reference material (TCP 121494–201403). As shown in figure 10, all of the raw materials and processed products were successfully analysed using the novel non-sequencing approach. Some of the highly processed products were not detected. This was likely due to the extensive processing that these products undergo. Species detection in the highly processed products may have been limited due to the processing as well as the presence of inhibitory ingredients present in these samples.

### 3.6. Application of PCR assay to commercial products

The ability of a novel non-sequencing approach to detect TCP and TC in commercial samples was tested with a variety of products, including raw materials, processed products and highly processed products (table 2). Overall, this method showed agreement as to the species detected in the products for 46 of the 64 samples (table 6). Of the 64 products analysed, all DNA extracted from the products was amplified by selected primers; two of these products declaring to contain TCP were amplified for *Trachemys scripta* DNA only. The result of commercial products is in good agreement with the self-made samples. For highly processed products, it is indicated that the specimens may differ in the degree of processing between different manufacturers and different batches from the same manufacturers. What is more, species detection in the processed products may have been limited due to the extensive processing that these products undergo as well as the presence of inhibitory ingredients present in these samples, or these could represent instances of mislabelling. More sensitive fluorescent dye or real-time PCR will be attempted in future research.

**Table 6.** Results of species identification in commercial samples. The results of PCR are reported as positive (+) or negative (−).

		PCR results							PCR results				
code	ingredients on label	CR	TS	MS	PS	AF	code	ingredients on label	CR	TS	MS	PS	AF
raw materials													
MG1	*Chinemys reevesii*	**+**	−	−			MB1	*Pelodiscus sinensis*				**+**	−
MG2	*Chinemys reevesii*	**+**	−	−			MB2	*Pelodiscus sinensis*				**+**	−
MG3	*Chinemys reevesii*	**+**	−	−			MB3	*Pelodiscus sinensis*				**+**	−
MG4	*Chinemys reevesii*	**+**	−	−			MB4	*Pelodiscus sinensis*				**+**	−
MG5	*Chinemys reevesii*	**+**	−	−			MB5	*Pelodiscus sinensis*				**+**	−
MG6	*Chinemys reevesii*	**+**	−	−			MB6	*Pelodiscus sinensis*				**+**	−
MG7	*Chinemys reevesii*	−	**+**	−			MB7	*Pelodiscus sinensis*				**+**	−
MG8	*Chinemys reevesii*	−	**+**	−			MB8	*Pelodiscus sinensis*				**+**	−
MG9	*Chinemys reevesii*	**+**	−	−			MB9	*Pelodiscus sinensis*				**+**	−
MG10	*Chinemys reevesii*	**+**	−	−			MB10	*Pelodiscus sinensis*				**+**	−
processed products													
RG1	*Chinemys reevesii*	**+**	−	−	−	−	RB1	*Pelodiscus sinensis*	−	−	−	**+**	−
RG2	*Chinemys reevesii*	**+**	−	−	−	−	RB2	*Pelodiscus sinensis*	−	−	−	**+**	−
RG3	*Chinemys reevesii*	**+**	−	−	−	−	RB3	*Pelodiscus sinensis*	−	−	−	**+**	−
RG4	*Chinemys reevesii*	**+**	−	−	−	−	RB4	*Pelodiscus sinensis*	−	−	−	**+**	−
RG5	*Chinemys reevesii*	**+**	−	−	−	−	RB5	*Pelodiscus sinensis*	−	−	−	**+**	−
RG6	*Chinemys reevesii*	**+**	−	−	−	−	RB6	*Pelodiscus sinensis*	−	−	−	**+**	−
RG7	*Chinemys reevesii*	**+**	−	−	−	−	RB7	*Pelodiscus sinensis*	−	−	−	**+**	−
RG8	*Chinemys reevesii*	**+**	−	−	−	−	RB8	*Pelodiscus sinensis*	−	−	−	**+**	−
RG9	*Chinemys reevesii*	**+**	−	−	−	−	RB9	*Pelodiscus sinensis*	−	−	−	**+**	−
RG10	*Chinemys reevesii*	**+**	−	−	−	−	RB10	*Pelodiscus sinensis*	−	−	−	**+**	−
highly processed products													
PG1	*Chinemys reevesii*	−	−	−	−	−	PB1	*Pelodiscus sinensis*	−	−	−	**+**	−
PG2	*Chinemys reevesii*	−	−	−	−	−	PB2	*Pelodiscus sinensis*	−	−	−	−	−
PG3	*Chinemys reevesii*	−	−	−	−	−	PB3	*Pelodiscus sinensis*	−	−	−	**+**	−
PG4	*Chinemys reevesii*	−	−	−	−	−	PB4	*Pelodiscus sinensis*	−	−	−	−	−
PG5	*Chinemys reevesii*	−	−	−	−	−	PB5	*Pelodiscus sinensis*	−	−	−	−	−
PG6	*Chinemys reevesii*	−	−	−	−	−	PB6	*Pelodiscus sinensis*	−	−	−	−	−
PG7	*Chinemys reevesii*	−	−	−	−	−	PB7	*Pelodiscus sinensis*	−	−	−	−	−
PG8	*Chinemys reevesii*	−	−	−	−	−	PB8	*Pelodiscus sinensis*	−	−	−	−	−
PG9	*Chinemys reevesii*	−	−	−	−	−	PB9	*Pelodiscus sinensis*	−	−	−	**+**	−
PG10	*Chinemys reevesii*	−	−	−	−	−	PB10	*Pelodiscus sinensis*	−	−	−	**+**	−
PG11	*Chinemys reevesii*	**+**	−	−	−	−	PB11	*Pelodiscus sinensis*	−	−	−	−	−
PG12	*Chinemys reevesii*	**+**	−	−	−	−	PB12	*Pelodiscus sinensis*	−	−	−	−	−

## 4. Conclusion

In conclusion, a novel non-sequencing approach used for TCP and TC established here is simple, time-saving, low-cost, accurate and sensitive, although a validation step for amplicon sequencing may be needed to ensure accuracy in practice. Our technique could be important from an economic point of view in terms of fair trade and consumer rights and will be very useful for the inspection of edibility and medicinal TCM of TCP and TC.

## Data accessibility

This article does not contain any additional data.
